# An eConsultant versus a hospital-based outpatient consultation for general (internal) medicine: a costing analysis

**DOI:** 10.1186/s12913-023-09436-1

**Published:** 2023-05-11

**Authors:** Jenny Job, Caroline Nicholson, Maria Donald, Claire Jackson, Joshua Byrnes

**Affiliations:** 1 UQ-MRI Centre for Health System Reform and Integration, The University of Queensland, Level 8, Health Sciences Building Royal Brisbane and Women’s Hospital Campus, Brisbane, QLD 4029 Australia; 2 General Practice Clinical Unit, Faculty of Medicine, The University of Queensland, Brisbane, Australia; 3 General Practice and Primary Care Research, The University of Queensland, Brisbane, Australia; 4 Centre for Applied Health Economics, Health Economics School of Medicine and Dentistry, Griffith University, Brisbane, Australia

**Keywords:** eConsultant, eConsult, Outpatients, Costing, efficiency

## Abstract

**Background:**

The eConsultant model of care is an outpatient substitution approach which has been evaluated and implemented extensively internationally. It provides an asynchronous, digital, clinician-to-clinician advice service, giving primary care physicians remote access to specialist support for patient care within 3 business days. Results from initial trials of the eConsultant model in Australia support international evidence of reduced wait times and improved access to specialist input, avoidance of face-to-face hospital outpatient visits, and better integrated care. This study compared the cost of delivery of an eConsultant episode of care with that of a hospital-based outpatient appointment.

**Methods:**

A cost-minimisation analysis, using a decision analytic model, was used to compare the two approaches. eConsultant costs were calculated from specialist reported data (minutes spent preparing the response; the number of patients referred subsequently for a hospital-based outpatient appointment) and administration staff data (time spent recording the occasion-of-service). Outpatient costs were calculated using finance data and information from outpatient clinic managers at the hospital-based outpatient clinic. The primary outcome was incremental cost saving per patient from a hospital system perspective. Uncertainty was explored using one-way sensitivity analyses and characterised with probabilistic sensitivity analysis using 10,000 Monte Carlo simulations.

**Results:**

The traditional referral pathway cost estimate was $587.20/consult compared to $226.13/consult for an eConsultant episode: an efficiency saving of $361.07 per patient. The incremental difference between eConsultant and traditional care was most sensitive to the cost estimate of an outpatient attendance, the time for a specialist to complete an eConsult, and the probability of a patient requiring a face-to-face hospital-based attendance following an eConsult. However, at the upper bounds of each of these estimates, an eConsult remained the most cost-efficient model. In 96.5% of the Monte Carlo simulations eConsult was found to be more cost efficient than the traditional approach.

**Conclusions:**

The eConsultant model of care was associated with a 61.5% efficiency gain, allowing diversion of support to hospital-based outpatient appointments.

**Supplementary Information:**

The online version contains supplementary material available at 10.1186/s12913-023-09436-1.

## Introduction

Excessive wait times for specialist outpatient appointments are a significant problem facing health systems internationally and have increased substantially in the last decade [1]. Lack of timely access to specialist care has been linked to inefficiencies in health care delivery, deterioration in health and dissatisfaction [2]. In line with other countries, Australia’s ageing population, and the subsequent growth in rates of chronic, complex disease, are placing increasing demand pressures on the health system in a time of significant fiscal constraints. As a result, some patients wait longer than they should for a specialist outpatient appointment, with public hospital non-urgent cases being seen outside clinically recommended times and the number of long waits steadily increasing since 2017 [3]. This has been exacerbated in recent years with COVID-19 placing further strain on the health system. In addition, approximately 28% of the Australian population live in rural and remote areas [4]. These Australians face unique challenges due to their geographic location and often have poorer health outcomes than people living in metropolitan areas [5]. People living in rural and remote areas have poorer access to health care services and accessing specialist services takes significant time and cost to attend outpatient appointments [5].

Outpatient services are resource intense and there has been a call for realigning resources and improved processes in outpatient services [3, 6]. In addition, there is a recognition that patients with complex, chronic diseases require ongoing team management co-ordinated by the primary care physician/general practitioner (GP) who can provide continuity of care in partnership with other health providers [7].

The eConsult model of care is an outpatient substitution approach which has been evaluated, and implemented extensively internationally [8–11]. It provides an asynchronous, digital, clinician-to-clinician advice service, giving GPs remote access to specialist support for patient care within 3 business days [8]. Findings from initial evaluations of Australian eConsultant services support international evidence of reduced wait times and improved access to specialist input, significant avoidance of face-to-face hospital outpatient visits and better integrated care. [12, 13]. Additionally, eConsultant offers workplace flexibility for the specialist, with the ability to deliver advice at the most suitable time and place. Our eConsultant service was piloted and implemented with a general (internal) medicine physician specialist, able to provide advice across all medical subspecialities bar dermatology [14]. The specialist was employed by an urban tertiary hospital which provided hospital-based general medicine clinics. In 2019, the hospital complemented this with an eConsultant service to two Australian regions (Western Queensland, and Brisbane South), resulting in 87% of requests for advice to the eConsultant replacing a traditional outpatient referral. This paper compares the cost of delivery of a traditional hospital-based outpatient appointment with that delivered via an eConsultant service. It was hypothesised that eConsultant would be more efficient to deliver.

## Methods

A cost-minimisation analysis from the hospital service provider perspective was used to estimate the incremental cost per patient of an eConsult compared with the traditional referral pathway, with a year time horizon. All costs are reported in 2020/21 in Australian dollars and discounting of future costs was not applied, appropriate to the time horizon. The resources and costs to deliver the eConsultant service was compared with the cost of delivery of a traditional hospital-based general medicine outpatient appointment for the same clinical patient-population.

### Study setting

The Mater Hospital South Brisbane (Mater) is a large urban hospital which provides seven half-day general medicine outpatient clinics per fortnight. In 2020–2021, the hospital implemented an eConsultant service with 15 general practices in Western Queensland and Brisbane South to investigate opportunities to safely substitute traditional face-to-face outpatient care.

The general medicine outpatient clinics are funded by Queensland Health, and the eConsultant service is funded by the Queensland eConsultant Partnership Program (QePP) at the specialist’s current sessional appointment. The eConsultant works remote to the hospital.

### The traditional outpatient service

GPs send a patient referral for a traditional outpatient appointment to the Mater Referral Management Centre (RMC) (Additional File 1). Referrals are checked for mandatory requirements (i.e., patient details) and printed (the KPI for this step is 24 hours from receipt of referral by the RMC). The referral is then reviewed by a RMC nurse to ensure that (1) the minimum data set has been received, (2) the referral is mapped to the correct speciality, and (3) the service is available. The RMC nurse may be required to re-review the referral if more information is subsequently received. An administration staff member then updates the patient record or registers the patient, and the GP is sent a receipt of the referral and a request for more information if required. A registrar or Visiting Medical Officer then categorises the patient based on urgency (1,2 or 3) or declines the referral. In Queensland, there are three outpatient urgency categories with recommended timeframes for consultation of within 30 days of being added to the outpatient wait list for Category 1 (Urgent); within 90 days for Category 2 (Semi-urgent) and within 365 days for Category 3 patients (Non-urgent) [15].

Once categorised, an administration officer updates the patient record with the category (the KPI for this step is 5 days from receipt of the referral by the RMC). The referral is then registered and waitlisted by the appointment management and call centre team. Face-to face, telehealth and telephone consults are conducted from the clinic. Wait time calculations starts when a referral is marked as delivered, that is, a referral is seen by the RMC and finishes once the patient is seen by the specialist. The specialist dictates a discharge letter post consultation which is typed and sent to the GP by an Administration Officer (AO) in the front desk team.

### The eConsultant service

**Fig. 1:**
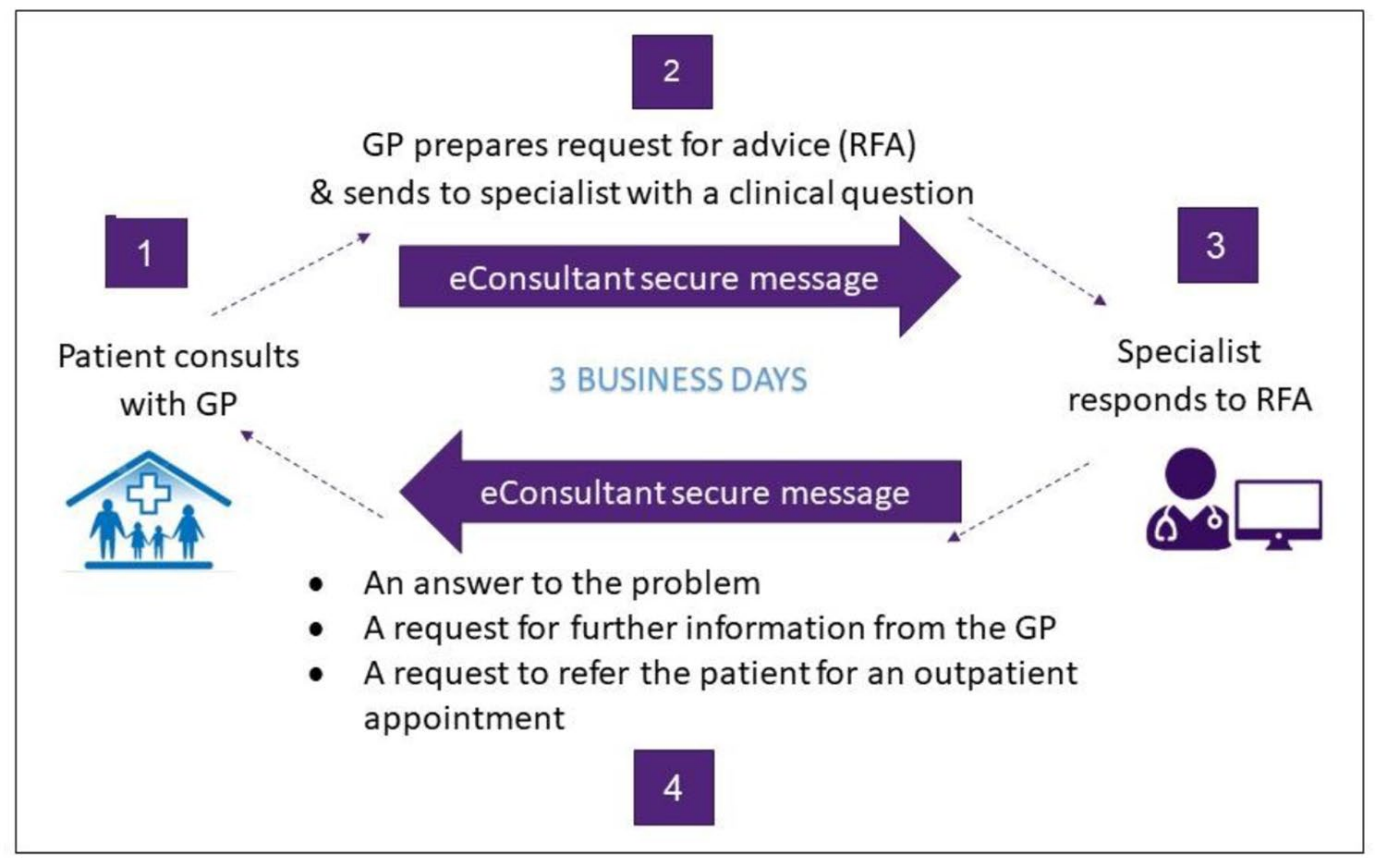
The eConsultant service

GPs enrolled in the eConsultant service have the option to send a Request for Advice (RFA) to the eConsultant for patients (category 1–3) who would normally be referred for a traditional outpatient appointment. GPs send a RFA, using a template, to the specialist (the eConsultant), with supporting information auto-populated from the patient’s record via the GP’s clinical information system. The RFA must include a specific question/s. The eConsultant replies within 3 business days with an answer to the problem; a request for further information; or a recommendation that the patient is referred for a traditional outpatient appointment (Fig. 1).

The eConsultant receives an email notification of a RFA and logs on to a web-based portal to access and reply to the RFA. This portal can be accessed from any location from a portable device or computer. An AO registers the patient (if not already registered) and records the RFA response in the patient chart.

The GP receives a documented record of the eConsultant advice via secure messaging to the practice inbox. All treatment decisions are made in partnership with the patient, and on the understanding that there is the option for a usual care specialist referral. The GP has the option to send additional follow-up RFAs about the same patient. GPs use the same billing practice as they would for a regular consultation. Audit via an independent physician ensures RFA fall within appropriate categorisation for outpatient referral.

### Data sources

A 12-month retrospective review of patient activity data for the period 2020–2021 was conducted. Costing data was collected from the outpatient service (14 clinics per month, excluding young adult and perioperative patients) and compared to data collected for the eConsultant service.

Hospital-based general medicine outpatient clinic consultations included in person, telehealth provider and telephone delivered consultations. Telehealth provider consultations are via video and are face time appointments where the specialist is located at the hospital and the patient is located off site. Telephone appointments are conducted by the specialist with the patient over the phone. Peri-operative and young adult hospital-based outpatient clinic consultations were not included as these are not covered by the eConsultant. A nurse and an administration person are always assigned to a general medicine clinic (even if no other specialists are there at that time) and will be shared with other clinics running at the same time.

Out-patient cost per attendance data were provided to the researchers by the hospital informatics team along with service data if the attendance was for a new or review episode, and if the attendance was face-to-face, telehealth or via telephone. Costs pertaining to the out-patient care having been attributed to each attendance using standardised managerial accounting practices by accredited hospital staff [16]. Managerial practices automatically assign costs based on a 30 min presentation for new patients. However, an audit of the new consultations (n = 94) over this period identified the actual mean time for new face-to-face appointments was 41 (SD 14.7) minutes. As such, the cost of a face-to-face outpatient visit was scaled up using a factor of 1.36 (41/30). Costs included labour (administration and clinician including allowances), direct supplies and oncosts (Additional file 2). The same cost for face-to-face outpatient attendances was used for both the traditional model as well as for those patients for whom a face-to-face attendance had been requested following an eConsultation. As digital infrastructure and secure messaging software are required by both the hospital-based outpatient and eConsultant services the costs were not included in the analysis.

For the eConsultant service, the cost for new and review patients was based on the time for the administrative officer and specialist. For the administrative officer, data on time estimates was derived for both new and review patients separately, considering the longer time associated with first establishing a patient within the system. For the specialist, data on time estimates were sourced from the specialist’s logged records for each RFA response. Specialist time was derived separately for instances that subsequently resulted in a request for a face-to-face attendance and those that did not for both new and review patients. This reflects that new patients are likely to take longer than review patients and those that require face-to-face attendance may take less time in completing the initial eConsultation. The cost per staff minute were based on Mater award pay levels provided by the hospital. The administrative officer was based on a level MCA3 and assumes no shift or overtime penalties apply based on the hours of operation of the clinic. The specialist’s pay was based on a L24 M02-3 specialist and includes professional allowances. For both the administrative officer and specialist, pay levels for 2020–2021 were used and oncosts were estimated based on advice from the Mater Business and Finance of a 30% mark up on salaries. Although, general practitioners (n = 54) reported taking the same time to complete an RFA as doing a traditional referral (mean: 13.84 min, SD:8.66); this cost and the cost of follow up attendances with general practitioners was excluded as they are health care services provided in the community (and not included in a hospital health service provider perspective). Time estimates included eConsultant post consult administration and evaluation.

As a hospital health service provider perspective was adopted, the cost for an outpatient attendance in which a patient does not attend was costed equal to that of one in which a patient attends. This reflects the opportunity cost of the resources (including clinician time) that has been allocated to that scheduled appointment which cannot otherwise be redirected. The probability of a patient not attending a scheduled face-to-face appointment was assumed to be 6% based on the measure for the overall outpatient clinic at the hospital. The cost of outpatient attendances subsequent to an eConsult service were assumed to be equal to that of a face-to-face outpatient attendance through the traditional referral pathway. Specifically, this is considered as: Cost of outpatient attendance subsequent to an eConsult = outpatient face-to-face attendance x ƛ where ƛ is a scale variable set to 1 in the basecase.

The proportion of new patients, compared to review patients, who received an eConsult was 96.2%. The probability that an eConsult would result in a subsequent face-to-face outpatient attendance was estimated for both new and review patients based on the specialist’s logged data. For new patients, a subsequent face to face attendance was required in 15.2% of cases (19/125), and 14.3% for review patients (1/7). For the traditional model, the probability that an outpatient attendance was conducted face to face, via telehealth or telephone was estimated for both new and review patients. For new patients, face to face attendances were conducted in 77.9% of cases (106/136), and for those that did not require a face-to-face attendance, 6.7% (2/30) were conducted via telehealth and 93.3% (28/30) via telephone. For review patients, face to face attendances were conducted in 84.9% of cases (298/351), and for those that did not require a face-to-face attendance, 7.5% (4/53) were conducted via telehealth and 92.5% (49/53) via telephone.

### Analytical approach

**Fig. 2:**
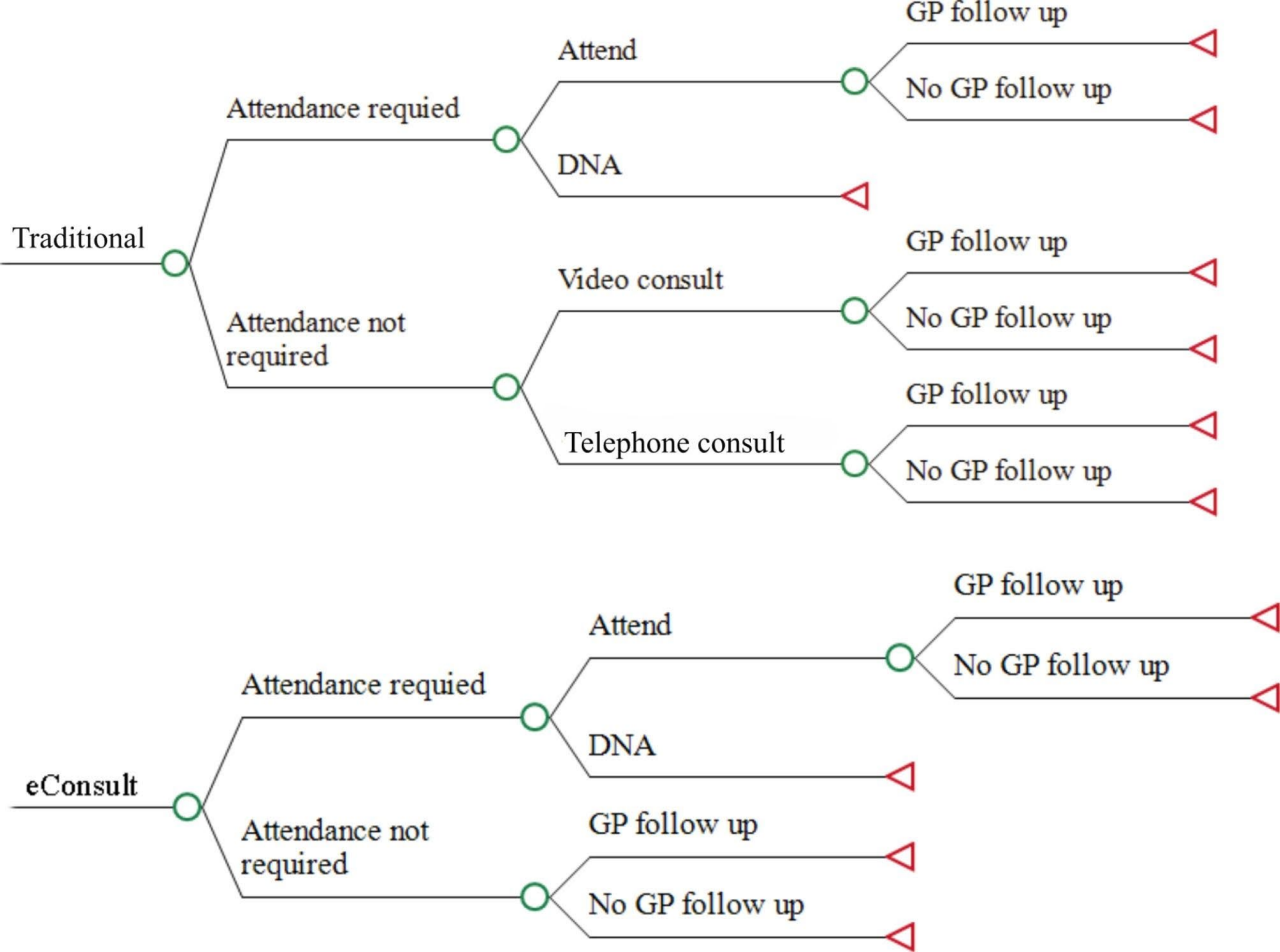
Decision analytic model

A decision analytic model was constructed in TreeAge Pro and graphically presented in Fig. 2 [17]. The expected cost per patient for both an eConsult and traditional outpatient appointment are estimated separately by multiplying the alternative specific conditional cumulative probabilities of each pathway and the cost of that pathway. Input parameters for the model are provided in Table 1. The primary outcome from the model is the incremental cost per patient for eConsult compared to the traditional outpatient referral pathway. Uncertainty was explored using one-way sensitivity analyses and characterised with probabilistic sensitivity analysis using 10,000 Monte Carlo simulations. For each Monte Carlo simulation an estimate for each of the models’ input parameters is drawn from a distribution. The distribution type and parameters are provided in Table 1. Beta distributions were used for transition probabilities, log normal distributions for count estimates (time), normal distribution for staff salary estimates and gamma distributions for outpatient clinic attendances. Distribution parameters were estimated based on (1) the data collected within the evaluation except for staff salary, for which the standard deviation was assumed to be 10% of the mean and the probability of a patient not attending a scheduled face to face attendance which assumed an alpha and beta of 6 and 94 (i.e., 6 / (6 + 94) = 6%) respectively and (2) an assumed normal distribution for the scale variable applied to the cost of an outpatient attendance subsequent to an eConsult with a standard deviation of 0.25.

**Table 1:** Input parameters and distribution

Input	Base case estimate	Distribution	Distribution parameter(s)
**eConsult**			
Transition probabilities (%)			
New patient	96.2%	Beta	ɑ: 125; β:5
DNA face to face attendances	6%	Beta	ɑ: 6; β:94
Subsequent face to face attendance			
New patients	15.2%	Beta	ɑ: 19; β:106
Review patients	14.3%	Beta	ɑ: 1; β:6
Time for eConsult			
Administration (min)			
New patient	20	Gamma	ɑ: 16.00; ƛ:0.8
Review patient	10	Gamma	ɑ: 16.00; ƛ:1.6
Specialist time (min)			
- Subsequent face to face attendance			
New patient	16.32	Gamma	ɑ: 1.60; ƛ:0.09
Review patient	8.00	Gamma	ɑ: 1.78; ƛ:0.22
- No subsequent face to face attendance			
New patient	28.82	Gamma	ɑ: 2.71; ƛ:0.09
Review patient	13.00	Gamma	ɑ: 22.53; ƛ:1.73
Cost for staff ($/min)			
Administration officer	0.72	Normal	µ: 0.72;σ: 0.07
Specialist	3.59	Normal	µ: 3.59;σ: 0.36
Subsequent outpatient clinic cost			
Scale relative to traditional pathway	1.00	Normal	µ: 1.00; σ: 0.25
**Traditional**			
Transition probabilities (%)			
Face to face attendance			
New patient	77.9%	Beta	ɑ: 106; β:30
Review patient	84.9%	Beta	ɑ: 1; β:28
Video attendance			
New patient	6.7%	Beta	ɑ: 298; β:53
Review patient	7.5%	Beta	ɑ: 4; β:49
Outpatient clinic cost			
Face to face			
New patient	638.50	Gamma	ɑ: 3.835; ƛ:0.006
Review patient	319.69	Gamma	ɑ: 2.629; ƛ:0.008
Video			
New Patient	574.75	Gamma	ɑ: 1.061; ƛ:0.002
Review patient	276.07	Gamma	ɑ: 2.075; ƛ:0.008
Telephone			
New patient	282.61	Gamma	ɑ: 2.439; ƛ:0.008
Review patient	239.47	Gamma	ɑ: 2.821; ƛ:0.012

## Results

**Fig. 3:**
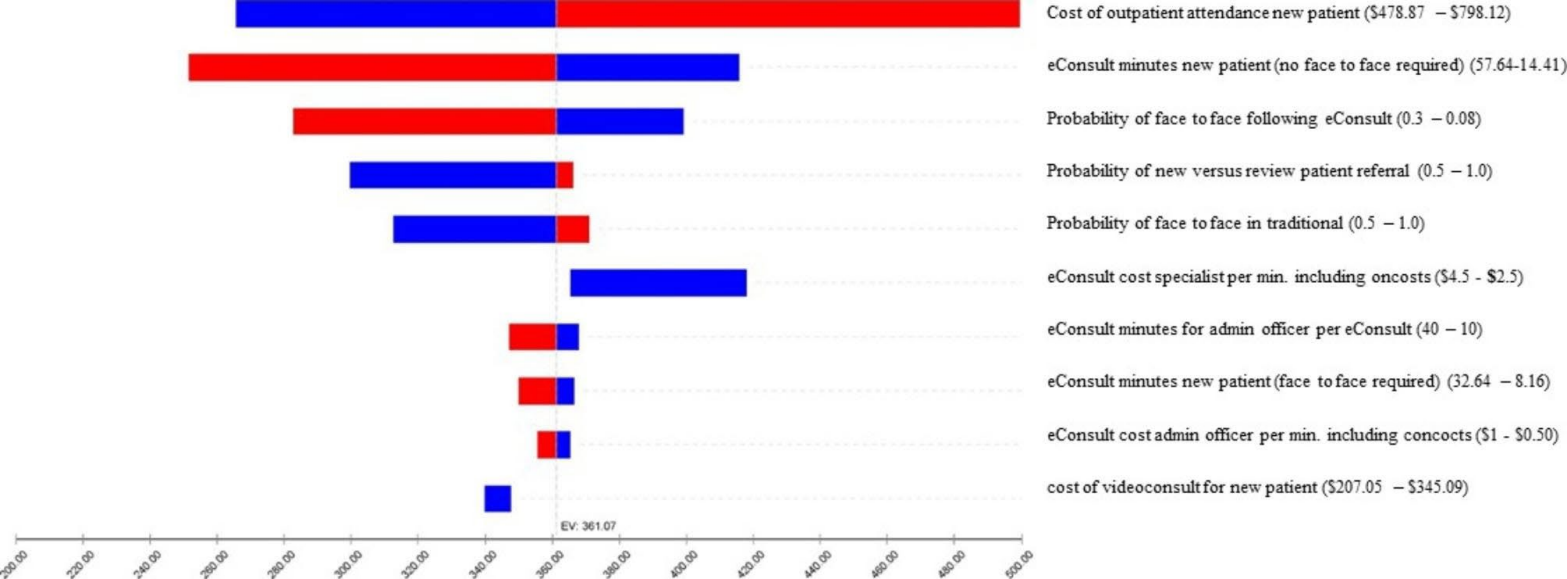
One-way sensitivity analysis

The traditional outpatient referral pathway cost estimate was $587.20 compared to $226.13 for an eConsult, an efficiency of $361.07 per patient (a 61.5% efficiency). Results from the one-way sensitivity analyses are presented in Fig. 3.

The incremental difference between eConsultant and traditional was most sensitive to the cost estimate of an outpatient attendance, the time for a specialist to complete an eConsult, and the probability of a patient requiring a face-to-face outpatient attendance following an eConsult. However, at the upper bounds of each of these estimates, an eConsult remained the most cost-efficient service. Increasing the cost of an outpatient attendance subsequent to an eConsult reduced the estimated difference between the eConsult and traditional referral pathways. However, the cost of a subsequent attendance would need to be 5.05 times the cost associated with an attendance via the traditional pathway for there to no longer be any difference in the expected costs between the two referral models.

**Fig. 4:**
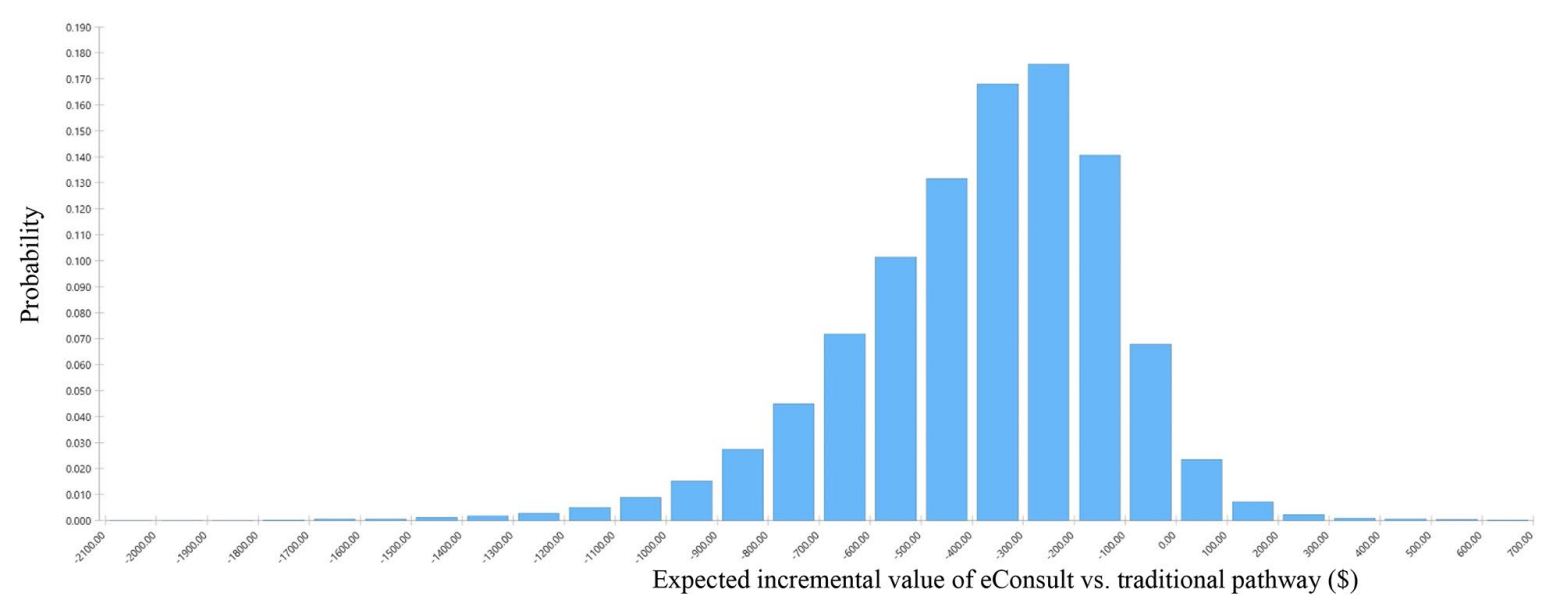
Probabilistic sensitivity analysis

Results from the probabilistic sensitivity analysis are presented in Fig. 4. In 96.5% of the Monte Carlo simulations an eConsult was found to cost less than the traditional approach (Fig. 4).

## Discussion

Post COVID-19 health systems internationally have been energised to the opportunities of digital health to improve access and cost of care. In a period of extreme pressure on wages growth, inflation and demand management, this paper demonstrates the efficiencies to be made within the Australian health context via addition of the eConsultant service as one of these platforms. The approach preserves the desired outcome for patient and GP – specialist support for decision-making and ongoing management – but improves access, timeliness, and efficiency in clinician-to-clinician linkage. It allows maximal allocation of scarce resources to clinical rather than administrative and support costs and frees clinicians to communicate at times convenient to their work schedules. Previous work has identified the small percentage (13%) of eConsultant RFA which require subsequent face-to-face outpatient assessment. This evaluation suggests eConsultant is associated with a 61.5% efficiency to the hospital when compared with hospital-based outpatient appointments, with much of the saving related to non-clinical costs.

The cost efficiency of the eConsultant service when compared to hospital-based outpatient consultations can be attributed to the cost savings due to low fixed costs and reduced clinician consulting time. Uncertainty in this analysis was principally due to the estimated cost of an outpatient attendance. The operational costs and labour for an outpatient clinic are substantial including infrastructure and information technology systems, electricity with its escalating price, and labour. Labour costs include a nurse who is allocated to a clinic regardless of the requirement for patient contact adding a substantial cost to the hospital-based models of care.

This is the first costing analysis of the eConsultant service in Australia. Our findings support international research that the model is safe and less costly. Depending on the impact approach, these studies have found eConsult services provide cost savings to the healthcare system, a return of investment, and societal savings when compared with hospital-based services [18–22]. Liddy’s group estimated total potential societal savings (included estimated direct costs to the payer and indirect costs to the patient) associated with their eConsult service to a remote Canadian community between August 2014 and April 2016 at $180,552.73 or $1,100.93 per eConsult [19]. Additional potential cost savings include the prevention of deterioration in patient health due to vastly reduced wait times for specialist input and effective treatment options being provided sooner, as well as more effective future specialist consultations if needed [23].

In addition to calculated efficiency from the hospital services perspective, eConsult services offer efficiency gains at the GP and specialist level. Previous advice options for general practitioners include phone calls to specialists, generally involving inefficient phone tag, an ad hoc advice option, and no formal documentation at the general practice or hospital service. In contrast, eConsultant is a documented advice service that operates asynchronously allowing busy clinicians to give and receive advice securely during clinical downtimes. Specialists can conduct eConsultant from home thus offering workplace flexibility and reduced travel time and costs for clinic attendance.

### Limitations

Although international evidence, all be it limited [8] has demonstrated that eConsults have the potential to improve health outcomes [10], this analysis is restricted in its comparison of hospital health service delivery costs. A full-economic evaluation, such as a cost-utlility analysis over an appropriate analytic time horizon that includes subsequent impact on health outcomes is required to address the value of eConsults. Overcoming previously identified challenges to evaluating impact on mortality and health related quality of life within a robust study design is critical [21].

Whilst the analysis outlined has excluded digital infrastructure, software, and capital costs such as building depreciation associated with healthcare delivery models, future analyses that explicitly consider these costs in providing capacity is warranted. GP time at follow-up appointments after an eConsult was not included in the analysis as they fell within usual follow up for patients requiring care modification. Future research however could include a sensitivity analysis for these costs. Concerns regarding inappropriate outpatient categorisation have been addressed by annual audit of eConsultant referrals by an external general medicine specialist – all have met Category 1–3 criteria.

The current service has delivered a 2-business day turnaround for the 15 participating practices. Scaling up without increasing wait time and preserving service integrity for hundreds of practices is the future challenge. Our partners in Ontario, have maintained this with over 100,000 consultations per year across 23 specialties [24]. Further costing analysis will be conducted with the addition of specialties including dermatology and endocrinology.

While this analysis looked at cost from a hospital system perspective, future research should include costs from a general practice and patient perspective. Additional savings to patients for an eConsult compared to face-to-face appointments can include avoidance of costs for travel, parking, and time off work for patients and their carers [23]. Patients from rural and remote areas often have additional costs for overnight accommodation close to the hospital outpatient service. For some patients several consultations may be required following investigations if a workup is not completed prior to an initial face-to-face appointment. In 2020–21 the Queensland Government allocated $94.8 million to support rural Queenslanders to travel to their appointments [25] and many patients still experience out of pocket expenses for travel for themselves and carers in addition to loss of income due to work leave.

The eConsultant model of care meets calls for primary care system reform that improves access, and supports continuity of care and integration of primary and secondary care by embracing and building on utilisation of digital technologies post COVID-19 [26]. By leveraging secure messaging this eConsultant service offers excellent cybersecurity. With estimates that healthcare contributes to 7% of Australia’s carbon emissions [27], eConsultant supports climate action goals by reducing fossil fuels required for unnecessary long-distance travel for face-to-face outpatient visits as well as hospital power consumption and cost.

## Conclusion

This analysis of the delivery cost of Australia’s first eConsultant service compared with traditional hospital-based outpatient care suggests a 61.5% efficiency. This is achieved by reducing labour and infrastructure costs, thus allowing diversion of scarce resources to situations where face-to-face visits are essential. This is in addition to the documented benefits of reduced wait times for specialist input, reduced requirements for long distance travel, and the associated cost and loss of work time for patients and their carers. Workplace flexibility for participating specialists and reduced carbon footprint are additional gains. Further research should investigate the costing impact of scaleup to additional specialities with expanded general practice participation as the model is offered more widely across Queensland. Scale-up nationally of this service would be facilitated by an integrated national digital health infrastructure, and could contribute to a more equitable, accessible, and efficient Australian health system.

## Electronic supplementary material

Below is the link to the electronic supplementary material.

Supplementary Material 1

Supplementary Material 2

## Data Availability

The datasets analysed for the current study are available from the corresponding author on reasonable request.

## List of abbreviations

AO: Administration Officer
DNA: Did not attend
GP: primary care physician/general practitioner
Mater: Mater Hospital South Brisbane
RFA: Request for Advice
RMC: Referral Management Centre
