# Supplementary material for: An eConsultant versus a hospital-based outpatient consultation for general (internal) medicine: a costing analysis
Source: BMC Health Serv Res. 2023 May 11;23:478. doi: 10.1186/s12913-023-09436-1 (PMC10174616; doi:10.1186/s12913-023-09436-1)
Supplement: Supplementary file 1 — Supplementary Material 1 [file 12913_2023_9436_MOESM1_ESM.pdf]

The Traditional Face-to-Face Outpatient Appointment Flow-Chart

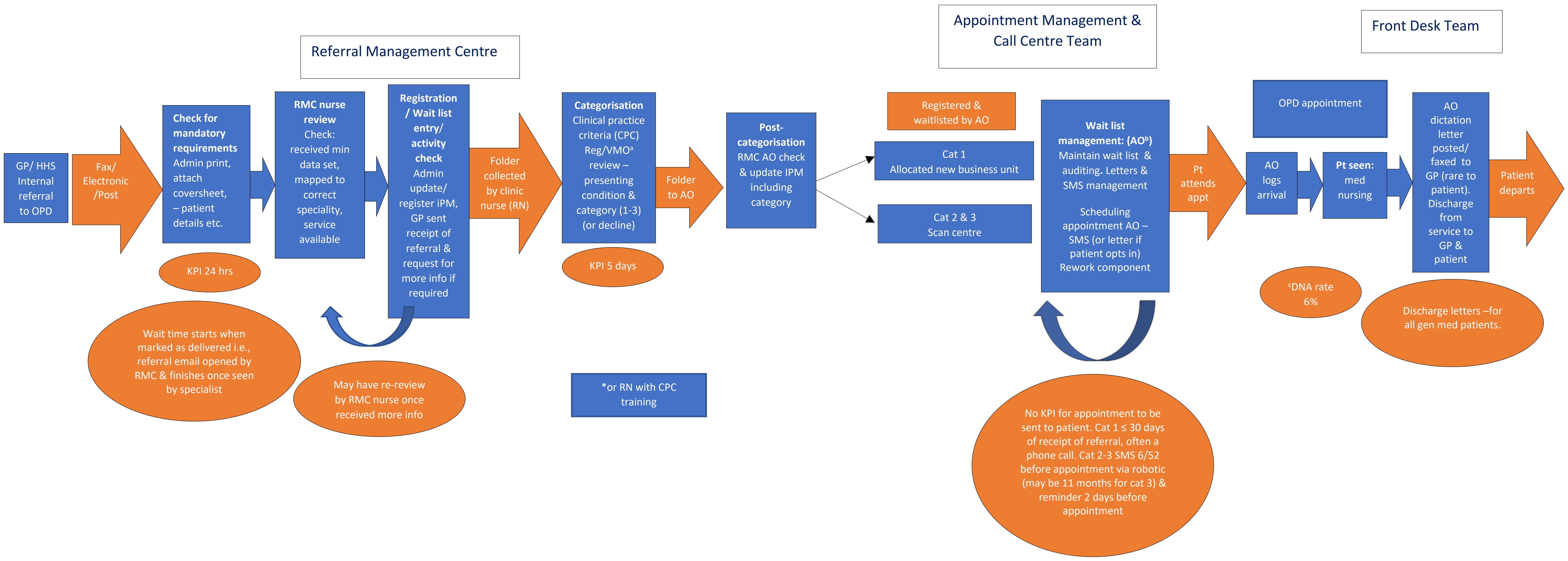

Queensland eConsultant Partnership Program Flow-Chart

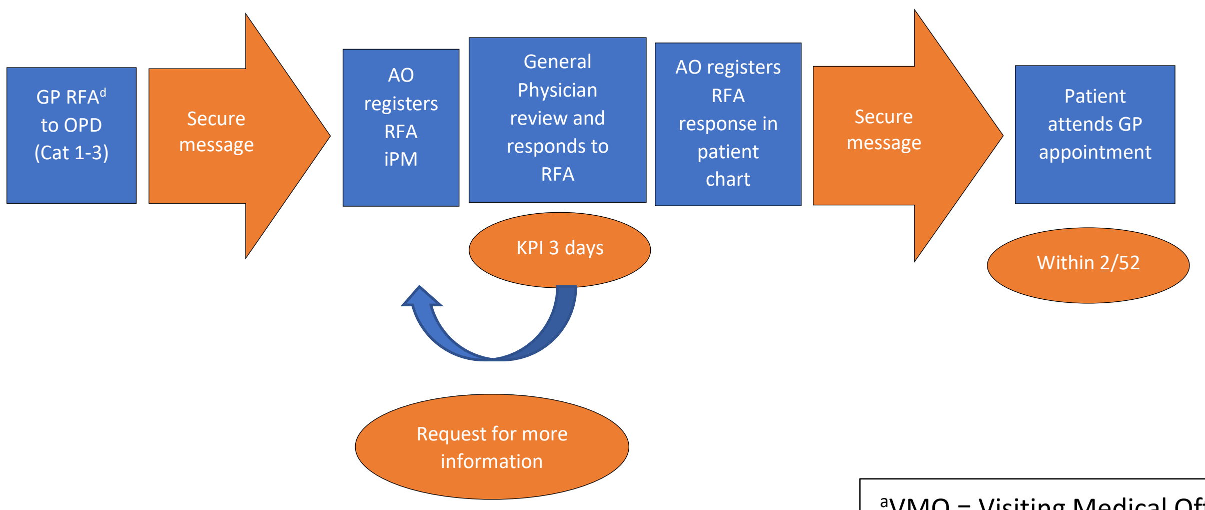

[a]VMO = Visiting Medical Office, [b]AO = Admin Office, [c] DNA = Did Not Arrive, [d]RFA = Request for Advice
