# Supplementary material for: An eConsultant versus a hospital-based outpatient consultation for general (internal) medicine: a costing analysis
Source: BMC Health Serv Res. 2023 May 11;23:478. doi: 10.1186/s12913-023-09436-1 (PMC10174616; doi:10.1186/s12913-023-09436-1)
Supplement: Supplementary file 2 — Supplementary Material 2 [file 12913_2023_9436_MOESM2_ESM.docx]

Additional File 2: Costs included and excluded from the analysis

| **Included** |  |
| --- | --- |
| VDS-MEDSUP-REBATE | Direct Supplies |
| VDS-DRUGS-REBATE | Direct Supplies |
| Variable Direct Supplies - Pathology | Direct Supplies |
| Variable Direct Supplies - Medical Supplies | Direct Supplies |
| Variable Direct Supplies - Imaging | Direct Supplies |
| Variable Direct Supplies - Hotel services | Direct Supplies |
| Variable Direct Supplies - Drugs | Direct Supplies |
| Outside Medical Fees | Direct Supplies |
| HPRAC-MGR | Labour - administration |
| Fixed Direct Other - Nurse | Labour - administration |
| Fixed Direct Other - EXCLUDED | Labour - administration |
| Fixed Direct Other - Administration | Labour - administration |
| Fixed Direct Other | Labour - administration |
| Fixed Direct Labour - Administration | Labour - administration |
| Variable Direct Other - Superannuation | Labour - all |
| Variable Direct Labour - Other Fee for service from invoice | Labour - all |
| Non Payroll Other Trade | Labour - all |
| Fixed Direct Other - Workers Compensation | Labour - all |
| Fixed Direct Other - Superannuation | Labour - all |
| Fixed Direct Other - Oncosts | Labour - all |
| Variable Direct Other - Staff Travel | Labour - clinical |
| Variable Direct Labour - Visiting Medical Officer | Labour - clinical |
| Variable Direct Labour - Other | Labour - clinical |
| Variable Direct Labour - Operations | Labour - clinical |
| Variable Direct Labour - Nurse | Labour - clinical |
| Variable Direct Labour - Medical | Labour - clinical |
| Variable Direct Labour - Health Practitioner | Labour - clinical |
| Medical Non Payroll | Labour - clinical |
| NURSE-MGR | Labour - management |
| MED-MGR | Labour - management |
| FDL-OPER-MGR | Labour - management |
| Variable Direct Other - Patient Travel Subsidy Scheme | Other |
| Variable Direct Other | Other |
| **Excluded** |  |
| Fixed Direct Facilities - Depreciation buildings | Building and facilities |
| Fixed Direct Facilities - Other | Building and facilities |
| Fixed Direct Equipment - Depreciation Other | Building and facilities |
| Fixed Direct Equipment - Other | Building and facilities |
| Fixed Indirect | Other |
| Hospital overhead | Other |
